# Chemical Composition, Algicidal, Antimicrobial, and Antioxidant Activities of the Essential Oils of *Taiwania flousiana* Gaussen

**DOI:** 10.3390/molecules25040967

**Published:** 2020-02-20

**Authors:** Hongmei Liu, Jiguang Huang, Sifan Yang, Jialin Li, Lijuan Zhou

**Affiliations:** 1 Key Lab of Natural Pesticides & Chemical Biology, Ministry of Education, South China Agricultural University, Guangzhou, Guangdong 510642, China; liuhongmei@stu.scau.edu.cn (H.L.); hnnyzx@scau.edu.cn (J.H.); 1945619358@stu.scau.edu.cn (J.L.); 2 Organic Agriculture, Wageningen University and Research, Gelderland, 6708 PB Wageningen, The Netherlands; sifan.yang@wur.nl

**Keywords:** *Taiwania flousiana* Gaussen, essential oil, algicidal activity, antimicrobial activity, antioxidant activity, algicidal mechanism

## Abstract

*Taiwania flousiana (T. flousiana*) Gaussen is a precious wood in the family Taxodiaceae. This study investigated the chemical components of the essential oil from the stem bark of *T. flousiana* and its algicidal, antifungal, and antioxidant properties. Sixty-nine compounds representing 89.70% of the stem bark essential oil were identified by GC-MS. The essential oil showed strong anti-algae, anti-bacteria, and anti-fungus activities against the tested species, and antioxidant activities. The IC_50_ values of the essential oil against chlorophyll a, chlorophyll b, and the total chlorophyll of *Spirogyra communis* (a species of algae), 24–96 h after the treatment, ranged from 31.77 to 84.92 μg/mL, while the IC_50_ values of butachlor ranged from 40.24 to 58.09 μg/mL. Ultrastructure changes revealed by the transmission electron microscopy indicated that the main algicidal action sites were the chloroplast and cell wall. The essential oil showed antifungal activities on *Rhizoctonia solani* (EC_50_ = 287.94 μg/mL) and *Colletotrichum gloeosporioiles* (EC_50_ = 378.90 μg/mL). It also showed bactericidal activities on *Ralstonia solanacearum* and *Staphylococcus aureus*, with zones of inhibition (ZOIs) being 18.66 and 16.75 mm, respectively at 40 μg/disk. Additionally, the essential oil possessed antioxidant activity estimated by 2,2-diphenyl-1-picrylhydrazyl (DPPH) method (IC_50_ = 33.51 μg/mL; IC_50_ value of the positive control ascorbic acid was 7.98 μg/mL). Thus, the essential oil of this plant might be used as a possible source of natural bioactive molecules in agrochemical industry as well as in food and cosmetic industries.

## 1. Introduction

*T. flousiana* (a synonym of *Taiwania cryptomerioides* Hayata), belonging to the family Taxodiaceae, is endemic to China. *T. flousiana* has been considered as one of the most precious woods in China for its outstanding quality. It has been used in construction industry, furniture industry, and paper industry [1,2,3]. Thus, the cultivation and plantation of *T. flousiana* have received significant attention [4,5,6,7,8]. The chemical constituents of the extract from the bark of *T. flousiana* have been investigated [9] and there were patents on the use of the chemicals from *T. flousiana* in medicinal industry [10]. Recently, a new chemical isolated from *T. flousiana* with strong herbicidal activity has been patented also [11]. However, it has not been previously investigated chemically for its essential oil.

In the production process of farm produce, various undesirable biotic factors such as algae and microbes can cause great loss of quantity and quality. *Spirogyra* (Zygnemataceae, Zygnematales) is a genus in the Class Zygnematophyceae (Conjugatophyceae), which is a member of the Infrakingdom Streptophyta. *Spirogyra communis* (Hassall) Kuetzing in the genus is widely distributed in freshwater habitats including flowing water, permanent ponds, and temporary pools and can cause great loss to farm produce [12,13]. Microbes such as *Rhizoctonia solani*, *Fusarium moniliforme* Sheld, and *Fusariun oxysporun*, etc., are common pathogens with a great diversity of host plants and can significantly reduce the quantity and quality of farm produce [14,15,16]. Normally, synthetic chemicals are extensively used to control them. However, the resistance issues caused by synthetic control agents are very common. Meanwhile, synthetic control agents lack selectivity and are toxic to non-target organisms. As an important part of pesticides, the algicide resistance has been reported. Meanwhile, synthetic algicides such as diuron are toxic and have negative impact on the environment. Similarly, synthetic antimicrobial chemicals have the same problems [17,18]. Therefore, as an alternative to these synthetic control agents, natural compounds and extracts from plants are the main sources [19]. Among them, essential oil has received significant attention [20,21].

Consumers need natural control agents not only in the production process of farm produce, but also in food preservation process. Nowadays, antioxidants have been widely used as food additives to provide protection against oxidative degradation of foods by free radicals. Normally, synthetic antioxidants such as butylated hydroxyanisole have been widely used. However, the toxicity of synthetic antioxidants has been questioned. Thus, the development of natural antioxidants is warmly desired [22]. Plant-derived essential oil has also received significant attention in this field [23]. In this work, we extracted the essential oil of *T. flousiana* and further (i) identified its chemical constituents; (ii) investigated its algicidal, antifungal, antibacterial, and antioxidant activities; (iii) characterized its mechanisms as an algicide.

## 2. Results

### 2.1. Chemical Components Identified in the Essential Oil

**Figure 1:**
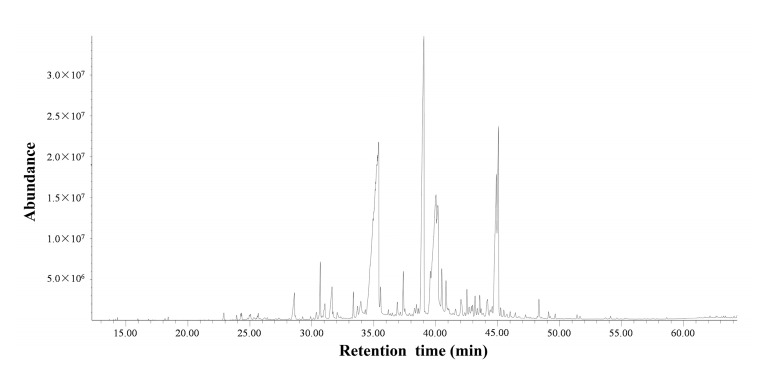
The GC-MS chromatogram of the essential oil.

The major components of the essential oil identified from *T. flousiana* are listed in Table 1. The yield of the essential oil extracted from *T. flousiana* was 0.31% (*v*/*w*). The GC-MS chromatogram of the essential oil is shown in Figure 1. In total, 69 components were identified and accounted for 89.70% of the total oil composition. The oil composition was dominated by the presence of hexadecanoic acid comprising 27.13% from total, followed by 2-penten-1-ol, 3-methyl-5-[octahydro-4, 5-dimethyl-7a-(1-methylethenyl)-1H-inden-4-yl]- (16.16%), linoleic acid (13.48%), podocarpa-6,8,11,13-tetraen-12-ol, 13-isopropyl-, acetate (7.56%), ferruginol (6.52%), and α-linolenic acid (5.41%). The contents of abietatriene, tetradecanoic acid, pentadecanoic acid ranged from 1.04 to 1.37%. The others were less than 1.00% (Table 1).

### 2.2. Algicidal Activity and Algicidal Mechanism of Action of T. flousiana Essential Oil

Algicidal activity of the essential oil extracted from stem bark of *T. flousiana* on *S. communis* was tested for the first time. The algicidal effects of *T. flousiana* essential oil on *S. communis* were dose-dependent at the concentrations from 12.5 to 200 μg/mL 24 to 72 h after the treatment.

The IC_50_ values of the essential oil on the inhibition of chlorophyll a ranged from 40.64 to 90.10 μg/mL 24–96 h after the treatment. As a contrast, those of butachlor ranged from 36.60 to 55.28 μg/mL. The IC_50_ values of the essential oil on the inhibition of chlorophyll b ranged from 53.39 to 106.91 μg/mL 24–96 h after the treatment. As a contrast, those of butachlor ranged from 47.29 to 79.12 μg/mL. Specially, 48 h after the treatment, The IC_50_ values of the essential oil was 47.49 μg/mL, while that of butachlor was 62.95 μg/mL, indicating that the essential oil showed a better algicidal effect at 48 h after the treatment based on the inhibition of chlorophyll b. The IC_50_ values of the essential oil on the inhibition of the total chlorophyll ranged from 31.77 to 84.92 μg/mL 24–96 h after the treatment. As a contrast, those of butachlor ranged from 40.24 to 58.09 μg/mL. Specially, 72 h after the treatment, the IC_50_ values of the essential oils was 31.77 μg/mL, while that of butachlor was 40.91 μg/mL, suggesting that the essential oil showed a better algicidal effect at 48 h after the treatment based on the inhibition of chlorophyll b.

In summary, the algicidal activity of the essential oil was comparable to or even better than that of butachlor (Table 2).

### 2.3. The Effect of Light on the Algicidal Activity of T. flousiana Essential Oil in S. communis

Further study revealed that light could affect the algicidal activity of *T. flousiana* essential oil in *S. communis*. 96 h after the essential oil treatment with light, the IC_50_ values of the essential oil ranged from 71.58 to 87.89 μg/mL, which were much lower than the values without light ranging from 1156.28 to 1229.24 μg/mL (Table 3). This result indicated that some active ingredients of the essential oil were photo-activated.

### 2.4. Antifungal Activities of T. flousiana Essential Oil

The effects of the essential oil of T. flousiana on Rhizoctonia solani Kuhn, Colletotrichum gloeosporioiles, Fusarium moniliforme Sheld, Thanatephorus cucumeris (Frank) Donk., Fusariun oxysporun f. sp. cubense, and Didymella bryoniae (Auersw.) Rehm. mycelial growth are listed in Table 4. The antifungal activity estimated by the EC_50_ value of the oil indicated a high variation of EC_50_ values among the fungal species (Table 4). The lowest EC_50_ value was observed against R. solani (287.94 μg/mL), while the highest was detected against D. bryoniae (3162.34 μg/mL). Generally, the oil showed better inhibitory activities on R. solani (287.94 μg/mL) and C. gloeosporioiles (378.90 μg/mL). The oil possessed activities on F. moniliforme, T. cucumeris, and F. oxysporun f. sp. cubense and the EC_50_ values were 923.03, 623.36, and 809.07 μg/mL, respectively.

### 2.5. Antibacterial Activities of T. flousiana Essential Oil

**Figure 2:**
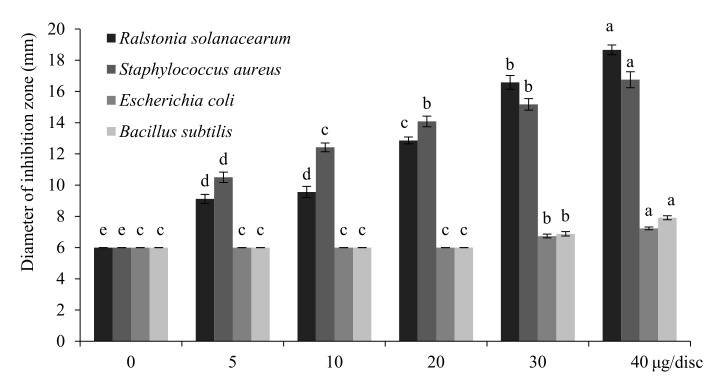
Antibacterial activity of *T. flousiana* essential oil estimated by diameter of inhibition zone. Diameter of inhibition zone includes diameter of discs (6 mm). Bacteria were cultured for 12 h at 37 °C. Different letters represent values that differed significantly in the Duncan’s multiple range test (*p* < 0.05).

The in vitro antibacterial activities of *T. flousiana* essential oil, against four species of microorganisms were estimated by measuring the diameter of inhibition zone and varied by the sample types and bacteria strains. The *T. flousiana* essential oil showed obvious activity against *Ralstonia solanacearum* Yabuuhi et al. (ATCC 11696) and *Staphylococcus aureus*, S. *aureus* (ATCC 25923) strains. The growth of the two bacteria species was inhibited by the essential oil in a dose-dependent manner under the exposure of increasing concentrations (0, 5, 10, 20, 30, and 40 μg/disk). At 40 μg/disk, the diameters of the inhibition zone (ZOI, mm) caused by the essential oil to *R. solanacearum* and *S. aureus* were 18.66 and 16.75 mm, respectively. However, the essential oil had not exhibited significant growth inhibition against *Escherichia coli* (Migula) Castellani and Chalmers (ATCC 8739) and *Bacillus subtilis* (Ehrenberg) Cohn. (ATCC 23857), with the ZOIs being 7.23 and 7.91 mm, respectively, at 40 μg/disk (Figure 2).

### 2.6. Antioxidant Activity

Essential oils have been proposed as potential substitutes for synthetic antioxidants in food preservation because of their antioxidant activity [44]. In this work, the antioxidant activity of *T. flousiana* oil was determined by DPPH (2,2-diphenyl-1-picrylhydrazyl) scavenging assay. In this assay, the antioxidant reacts with the stable free radical 2,2-diphenyl-1-picrylhydrazyl with a deep violet color and produces 2,2-diphenyl-1-picrylhydrazine with no color [45]. The free radical scavenging activity is usually expressed either as percentage of DPPH inhibition or by the antioxidant consumption for a 50% DPPH reduction (IC_50_). The amount of essential oil needed to decrease the initial DPPH by 50% (IC_50_) is a parameter widely used to measure the antioxidant activity. The lower the IC_50_ value, the more potent the antioxidant is. In our results, positive control ascorbic acid had the strongest antioxidant activity with IC_50_ value of 7.98 μg/mL. The IC_50_ value of the essential oil of *T. flousiana* was 33.51 μg/mL (Table 5), indicating it scavenged the free radical DPPH.

### 2.7. Effects of Essential Oil on Algal Internal Structure

**Figure 3:**
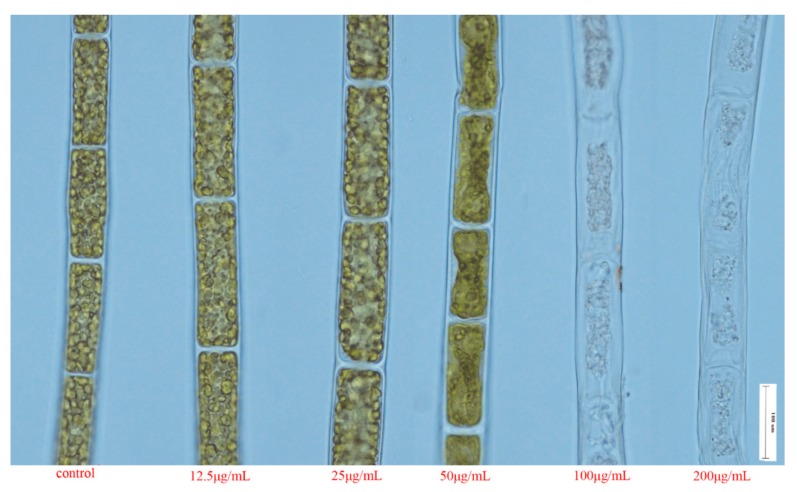
Effect of the essential oil of *T. flousiana* on the morphology of *S. communis*. *S. communis* was treated with the essential oil at different concentrations for 96 h, the morphological changes were imaged with microscope (Nikon Eclipse Ti) at a magnification of 10×.

We found that the chloroplast disintegration and the shrink of plane transverse cell walls became more apparent with the increase of the essential oil concentration. With the treatment of 50 μg/mL, the chloroplast started to disintegrate. Meanwhile the structure of the chloroplast shrank. Severe damage of cell structure was observed at higher essential oil concentrations (100–200 μg/mL). In these treatments, plasmolysis occurred and damage to basic cell structure was severe, as revealed by the fact that the chloroplast disintegration became more apparent, and as well by damage to the plasma membrane and was accompanied by increased cell wall opacity. As a contrast, intact cell wall and normally distributed chloroplast were present in the control cells (Figure 3).

### 2.8. Alteration of the Cell Ultrastructure of S. communis by the Essential Oil of T. flousiana

**Figure 4:**
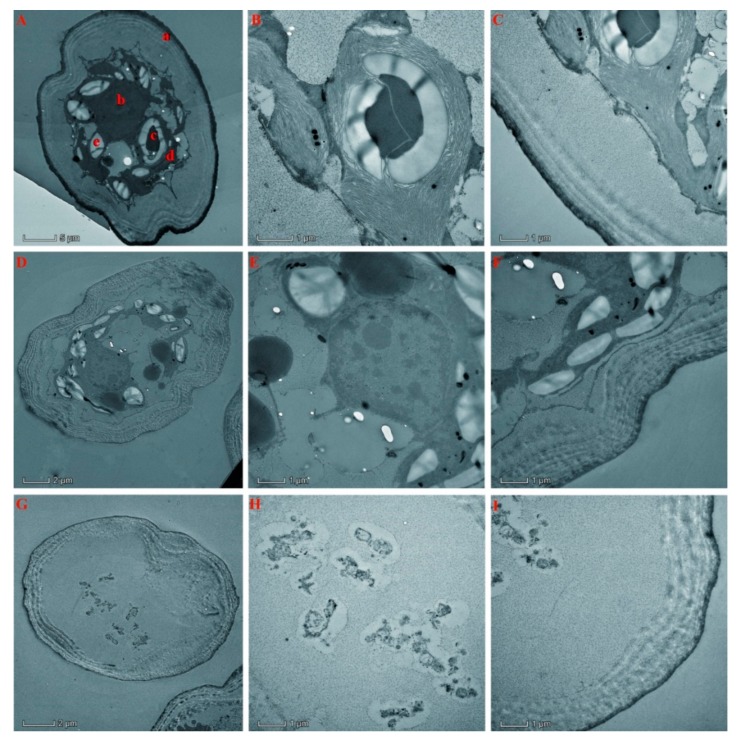
Effect of the essential oil of *T. flousiana* on the ultrastructure of *S. communis*. *S. communis* was treated with the essential oil at different concentrations (50 and 200 μg/mL) for 96 h and the ultrastructure changes were assessed by TEM). The control cells (**A**–**C**), treatment with the essential oil of *T. flousiana* (**D**–**F**) treated with the essential oil of *T. flousiana* at 50 μg/mL and (**G**–**I**) treated with the essential oil of *T. flousiana* at 200 μg/mL). a: cell wall; b: nucleus; c: chloroplast; d: thylakoid; e: starch grain.

The treated *S. communis* cells were analyzed by transmission electron microscope (TEM). Specifically, it was found that essential oil at 200 μg/mL could damage the cell wall and the chloroplast, leaving the cells vacuolated, with only some organelle remained in the cell (Figure 4G–I). This result indicated that the main action site of the essential oil of *T. flousiana* might be the cell wall and chloroplast. It was noteworthy that the chloroplast is an important target of the essential oil of *T. flousiana*, this finding further supported our result that the light contributed to the algicidal activity of the oils presented in Table 3.

## 3. Discussion

In 2001, research interests in the components of the essential oil of *T. cryptomerioides* were reported for α-cadinol, ferruginol, and cedrol isolated from the essential oils of sapwood and heartwood of *Taiwania cryptomerioides* Hayata [46], and a-cadinol, T-muurolol, ferruginol, and T-cadinol obtained from *T. cryptomerioides* heartwood [47]. Further study in 2012 identified 35 compounds from the twig essential oil of *T. cryptomerioides*, of which cadinol (45.9%), ferruginol (18.9%), and β-eudesmol (10.8%) were the major compounds [48]. In our study, 69 components were identified from the bark of *T. flousiana* and accounted for 89.70% of the total oil composition. The main components were hexadecanoic acid (27.13%), 2-penten-1-ol, 3-methyl-5-[octahydro -4,5-dimethyl-7a-(1-methylethenyl)-1H-inden-4-yl]-(16.16%), linoleic acid (13.48%), podocarpa- 6,8,11,13-tetraen-12-ol, 13-isopropyl-, acetate (7.56%), ferruginol (6.52%), and α-linolenic acid (5.41%). The contents of abietatriene, tetradecanoic acid, pentadecanoic acid ranged from 1.04 to 1.37%. The others were less than 1.00%. The diterpene ferruginol was also one of the major components of bark essential oil of *T. flousiana*, cedrol and β-eudesmol were also present in the bark essential oil of *T. flousiana*, but with quite less contents. The contents of ferruginol, cedrol, and β-eudesmol were 6.52%, 0.13%, and 0.06%, respectively. The differences of our results from those in the literatures were probably because of the different parts of the test material and its collection locations.

Further, we found that *T. flousiana* essential oil possessed inhibition of chlorophyll content on the *S. communis*, which is a species of algae. Harmful algae blooms have increased globally and many researchers are focusing on the development of the effective control agents. Plant-derived chemicals are important sources of selective and biodegradable algicides [49]. Recently, algicidal polyphenolic *p*-hydroxybenzoic acid, coumarin, and fatty acids have been isolated from different plants [50,51,52]. Wang et al. [53] reported the algicidal activities of essential oils from six plant species, namely, *Potamogeton cristatus*, *Potamogeton maackianus, Potamogeton lucens, Vallisneria spinulosa, Ceratophyllum demersum*, and *Hydrilla verticillata*. The inhibition rates of essential oils on *M. aeruginosa* were 30.2–41.7% at a concentration of 50.0 μg/mL. Phenolic and fatty acids were found to be the algicidal chemicals [54]. Normally, phenolic and fatty acids were the common components of essential oils, which provide useful information for further study of these chemicals in control of the submerged weeds. Notably, we found that light contributed to the algicidal activity of *T. flousiana* essential oil in *S. communis*. This indicated that some active ingredients of the essential oil were photo-activated, which deserves further study.

In 2017, Chen et al. reported that phytochemicals (ferruginol, T-cadinol, alpha-cadinol, and T-muurolol) of *T. cryptomerioides* heartwood had the potential to be used as environmentally benign antifungal agents against brown root rot fungus *Phellinus noxius* in place of synthetic or inorganic fungicides. Their results showed that ferruginol, T-cadinol, alpha-cadinol, and T-muurolol were found to exhibit excellent antifungal activities against *P. noxius*, with IC_50_ values 16.9, 25.8, 33.8 and 50.6 μg/mL, respectively [55]. In our study, the content of ferruginol in *T. flousiana* essential oil was 3.94% and the oil also showed antifungal activity. Thus, the antifungal activity of the active ingredient ferruginol deserves more attention.

In 2002, Wang et al. demonstrated that ferruginol exhibited the strongest antioxidant activity among the diterpenes isolated from *T. cryptomerioides* heartwood [56]. In 2012, Ho et al. also reported that *T. cryptomerioides* twig essential oil showed antioxidant activity against DPPH. The IC_50_ of the DPPH free radical scavenging capability of the essential oil was 90.80 μg/mL and ferruginol (IC_50_ = 48.0 μg/mL) was identified to be the main active ingredient for the free radical scavenging [57]. In our study, the content of ferruginol was 3.94% in *T. flousiana* oil while the IC_50_ value of the essential oil was 33.51 μg/mL. As a contrast, in the report of Ho et al. the content of ferruginol in *T. cryptomerioides* twig oil was 18.9% while the IC_50_ value of the essential oils was 90.80 μg/mL [57]. In general, the antioxidant activity of essential oils is the product of additive, synergistic, and/or antagonistic effects from a complex mixture of several classes of compounds. These suggested that other components of the *T. flousiana* essential oil may possess the antioxidant activity. The biological functions of each component of the essential oil need to be further investigated.

Additionally, our results indicated that the main action site of the essential oil of *T. flousiana* might be the cell wall and chloroplast. Mechanism study plays a very important role in the development of new algicidal chemicals. Further mechanism studies should focus on cell wall and chloroplast.

## 4. Material and Methods

### 4.1. Plant Material

Whole *T. flousiana* plant was collected from Enshi autonomous prefecture in central China in July, 2019 (Enshi, Hubei province, China). The plant was further identified as *T. flousiana* (a synonym of *Taiwania cryptomerioides* Hayata) and a voucher specimen was deposited in the Key Laboratory of Natural Pesticides & Chemical Biology, Ministry of Education, South China Agricultural University, China. The plant material was air-dried for up to 3 weeks at the ambient temperature and 24 h in a 50–60 °C incubator prior to pulverization.

### 4.2. Isolation of Essential Oil

The air-dried stem bark powder (250 g) of *T. flousiana* was subjected to hydrodistillation for 3 h using a Clevenger-type apparatus. The oil was dried with anhydrous sodium sulphate. The yield (v/w, dry weight basis) was calculated as volume (mL) of extracted essential oil per 250 g of plant material. Then, the essential oil was stored in hermetically sealed dark-glass at 4 °C until further analysis.

### 4.3. Analysis of the Essential Oil

Analysis of the essential oils was carried out with an Agilent Technologies 7693A Gas Chromatograph with 5977B Mass Spectrometer. A HP-5 MS capillary column (30 m × 0.25 mm × 0.25 μm; Agilent Technologies Inc., Santa Clara, USA) was employed. Analyses were carried out using helium as the carrier gas at a flow rate of 1.0 mL/min, split ratio: 15:1. Oven temperature was programmed as follows: 40 °C initially rising to 150 °C at a rate of 6 °C/min; rising to 270 °C at a rate of 3 °C/min; rising to 300 °C at a rate of 10 °C/min and held for 3 min. The injector and detector were held at 325 °C. The mixtures of the normal alkane of C_7_–C_30_ (1000 μg/mL) and EO samples dissolved in hexane of 0.8 μL were injected and all samples were filtered through a 0.22 μm organic phase filter. The Mass spectra were obtained by electron ionization (EI) at 70 eV, using a spectral range of 30–550 AMU in full scan mode. The MS (Agilent Technologies Inc., Santa Clara, USA) transfer line was set at 250 °C.

### 4.4. Identification of the Essential Oil Chemical Constituents

The essential oil constituents identification was carried out by comparing their recorded mass spectra with those stored in the National Institute of Standards and Technology Mass Spectral database (NIST 17 database) or with authentic compounds and confirmed by comparison of their retention index with authentic compounds reported in the literature [58]. The relative percent of each component in essential oil was counted by the area normalization method. The retention index was defined by the following:

RI = 100n + 100(t_x_ − t_n_)/(t_n + 1_ − t_n_) (1)

where t_n_, t_n + 1_, and t_x_ were net retention times [59].

Identification of the individual components was based on: (i) Comparison with the mass spectra of authentic reference compounds possible and by reference to NIST 17 database, and Adams terpene library [43]; (ii) comparison of their retention indices (RI) on a HP-5, calculated relative to the retention times of a series of C-7 to C-30 *n*-alkanes, with linear interpolation, with those of authentic compounds or literature data [43].

### 4.5. Evaluation of the Algicidal Activity with Light

*S. communis* was collected from the Southern China Botanical Garden, Chinese Academy of Sciences. *S. communis* was incubated in a modified Bold basal medium composed of NaNO_3_ (250 mg/L), K_2_HPO_4_ (75 mg/L), CaCl_2_·H_2_O (25 mg/L), MgSO_4_·7H_2_O (75 mg/L), NaCl (25 mg/L), KH_2_PO_4_ (175 mg/L), Na_2_EDTA·2H_2_O (4.5 g/L), FeCl_3_·6H_2_O (0.582 g/L), MnCl_2_·4H_2_O (0.246 g/L), ZnCl_2_ (0.030 g/L), Na_2_MoO4·2H_2_O (0.024 g/L), CoCl_2_·6H_2_O (0.012 g/L), vitamin B1 (1.1 g/L), vitamin B6 (0.025 g/L), and vitamin B12 (0.135 g/L) [60].

The alga was cultured in 500 mL of sterilized culture medium in 1000 mL conical flasks under an irradiance of 4000 lux, 12 h light/12 h dark (12:12), at 25 ± 1 °C for 5 days. Then the 10 mL of the algal cultures (0.1 g) was transferred to a 6-well plate. The stock solution of the essential oil was prepared in acetone. The final concentrations of the essential oil in the test solution were 12.5, 25, 50, 100, and 200 μg/mL, respectively. Acetone in the test solution was lower than 0.2% (v/v). The commercial herbicide, butachlor (12.5, 25, 50, 100, and 200 μg/mL), was used as a control. The plates were sealed with polyethylene wrapping film and incubated in a growth chamber at 25 ± 1 °C, RH 50–60%, and a photoperiod of 12:12. Each treatment has three replicates. All experiments were repeated at least three times.

The algicidal activity was determined by the chlorophyll content as described by Dere et al. [61] with some modifications. Specifically, after being dried with absorbent paper, *S. communis* in each replicate was grounded in 2 mL of 80% acetone. Then the mixture was transferred into a centrifugal tube (2 mL) followed by centrifugation for 10 min at 4000 rpm. The supernatant was transferred into a tube and diluted with acetone to 4 mL. Then 2 mL of the solution was mixed with 80% acetone to make a final volume of 10 mL and the absorbances were recorded at 663 nm and 645 nm. Acetone (80%) alone was used for the blank control. The amount of the pigments was calculated according to the following formulas:

C_a_ = 12.7 × A_663_ − 2.69 × A_645_ (2)

C_b_ = 22.9 × A_645_ − 4.68 × A_663_ (3)

C_ab_ = 8.02 × A_663_ + 20.21 × A_645_ (4)

The content of the chlorophyll was calculated according to the following formula:

The chlorophyll content (mg/g) = (Chlorophyll concentration × the volume of the tested

solution × the dilution factor)/the sample mass) × 100(5)

The inhibition rate of chlorophyll was calculated according to the following formula:

The inhibition rate of chlorophyll (%) = (([C] − [S])/[C]) × 100(6)

where [C] means the chlorophyll content of control and [S] means the chlorophyll content of sample.

### 4.6. Evaluation of the Algicidal Activity without Light

The same method as described above in 4.5 was followed except the plates were incubated under a photoperiod of 24:0 (dark:light). The inhibition rate of chlorophyll was evaluated 96 h after the treatment.

### 4.7. Morphological Changes of S. communis Treated with the Essential Oil of T. flousiana

The morphological changes of *S. communis* treated with the essential oil of *T. flousiana* for 24, 48, and 72 h were evaluated with light microscopy (Leica DMLB2, Leica Microsystems, Wetzlar, Germany). Transmission electron microscopy (TEM) (FEI Tecnai 12, FEI company, Hillsboro, USA) evaluation was further performed in order to examine the effect of the essential oil of *T. flousiana* (at 12.5, 25, 50, 100 and 200 μg/mL) on the ultra-structure of *S. communis*. The TEM samples were processed as previously described by Houot et al. [62]. Samples were examined by TEM (FEI Tecnai 12, FEI company, Hillsboro, USA).

### 4.8. Determination of Antimicrobial Effects of the Essential Oils on Mycelial Growth

Fungi including *R. solani*, *C. gloeosporioiles*, *F. moniliforme*, *T. cucumeris*, *F. oxysporun* f. sp. *cubense*, and *D. bryoniae* were obtained from the Department of Plant Pathology, South China Agricultural University. In vitro antifungal assays were conducted according to the method of Boubaker et al. [63], with slight modifications. Briefly, sterile molten potato-dextrose-agar (PDA) supplemented with essential oil of *T. flousiana*, at final concentrations of 62.5, 125, 250, 500, and 1000 μg/mL, was poured into Petri plates (6-cm-diameter). All tests were performed in PDA supplemented with 0.5% (*v*/*v*) DMSO to enhance oil solubility. Afterwards, plates were inoculated with fungal cultures with 5-mm diameter agar disks from one-week-old cultures, with mycelia surface facing down. The agar plates were then incubated at 25 °C for 2 days. Plates with medium supplemented with 0.5% DMSO only was used as the control. The antifungal activity was expressed as percent of mycelial radial growth inhibition and calculated according to the following formula: MGI (%) = ((C − T)/C) × 100, where C and T represent mycelial growth diameter in control and EO treated plates, respectively. Each treatment has three replicates. All experiments were repeated at least three times.

The in vitro antibacterial activity of the essential oil from *T. flousiana* was carried out by using filter paper disc diffusion assay [64,65]. Two Gram-positive bacteria *S. aureus* and *B. subtilis* and two Gram-negative bacteria *E. coli* and *R. solanacearum,* provided by the Department of Plant Pathology, South China Agricultural University, were tested. Typically, 500 μl of a suspension of the tested microorganisms (approx. 10^6^ colony-forming units (CFU)/mL) was spread with a sterile cotton swab on the surface of Mueller-Hinton agar (MHA) plates at 37 °C and allowed to dry for 10 min. A stock solution of the essential oil was prepared by dissolving 20 mg in 1 mL of DMSO. Then the stock solution was diluted with 0.1% aqueous Tween solution to get series solution of 4000, 3000, 2000, 1000, and 500 μg/mL. Each sterile filter paper disc (6 mm in diameter Whatman disks) was impregnated with 10 μL solutions, respectively. The Petri dishes were kept at 4 °C for 2 h to allow the diffusion of the oil, and further incubated at 37 °C for 24 h. Activity was expressed as percent of zone of inhibition (ZOI, mm). The net zone of inhibition was determined by subtracting the disc diameter (i.e., 6.0 mm) from the total zone of inhibition shown by the test disc in terms of clear zone around the disc. The control was the aqueous solution of 0.1% Tween + 8% DMSO. Each treatment had three replicates. All experiments were repeated at least three times.

### 4.9. Determination of Antioxidant Activity

The method of El-Gawad [66] was used. The antioxidant activity of *T. flousiana* essential oil was measured in terms of radical scavenging activity, using the stable radical DPPH (Sigma-Aldrich, Darmstadt, Germany) [48]. A reaction mixture of 1 mL of a hexane solution of the essential oil with different concentrations (5, 10, 20, 40, and 80 μg/mL) and equal volume of the methanolic solution of 0.3 mM DPPH was prepared, mixed well and incubated under dark condition for 15 min at room temperature. Ascorbic acid (5, 10, 20, 40, and 80 μg/mL) was used as the reference. The decrease in absorbance at 517 nm was determined using a spectrophotometer (UV-8500PC, Shanghai, China). The IC_50_ (the amount of sample necessary to decrease the absorbance of DPPH by 50%) was calculated graphically. Each treatment has three replicates. All experiments were repeated at least three times. The percent of the inhibition of the DPPH radical was calculated as following:

The inhibition (%) = 1 − (Absorbance of sample/Absorbance of control) × 100(7)

### 4.10. Statistical Analysis

Statistical analysis (ANOVA) by means of the IBM SPSS Statistics, Version 19.0 (International Business Machines Corporation, New York, USA) was applied to the data to determine the differences (*p* < 0.05). Then, the estimation of the median effective concentration (EC_50_), median inhibition concentration (IC_50_), and their 95% confidence limits were obtained. All quantitative data were presented as the mean ± SD of at least three independent experiments using the Duncan’s multiple range test or Student’s *t* test for group differences. A *p* < 0.05 was considered as statistically significant.

## 5. Conclusions

Current investigation highlights the detailed chemical composition of EO extracted from *T. flousiana* and their bioactive potential. Sixty-nine compounds representing 89.70% of the stem bark essential oil of *T. flousiana* were identified. The main components were hexadecanoic acid (27.13%), 2-penten-1-ol, 3-methyl-5-[octahydro-4,5-dimethyl-7a-(1-methylethenyl)-1H-inden-4-yl]- (16.16%), linoleic acid (13.48%), podocarpa-6,8,11,13-tetraen-12-ol, 13-isopropyl-, acetate (7.56%), ferruginol (6.52%), and α-linolenic acid (5.41%).

The algicidal activity of the essential oil on *S. communis* was comparable to that of butachlor. The IC_50_ values of the essential oil ranged from 31.77 to 84.92 μg/mL, while the IC_50_ values of butachlor ranged from 40.24 to 58.09 μg/mL. Thus the essential oil from *T. flousiana* could be considered as a potential algicidal substitute of synthetic ones. In addition, ultrastructure changes revealed by the transmission electron microscopy indicated that the main action sites of the essential oil of *T. flousiana* on *S. communis* cell were the chloroplast and cell wall.

The EO showed antifungal activities on *Rhizoctonia solani* (EC_50_ = 287.94 μg/mL) and *Colletotrichum gloeosporioiles* (EC_50_ = 378.90 μg/mL). Meanwhile, The EO also showed bactericidal activities against *Ralstonia solanacearum* and *Staphylococcus aureus*, with ZOIs being 18.66 and 16.75 mm, respectively at 40 μg/disk.

The level of antioxidant activity estimated by 2,2-diphenyl-1-picrylhydrazyl (DPPH) method showed that the essential oil of *T. flousiana* demonstrated obvious antioxidant activity with IC_50_ value of 33.51 μg/mL. While the IC_50_ value of the positive control ascorbic acid was 7.98 μg/mL. Thus, the essential oil of this plant could be used as a potential source of natural bioactive molecules in agrochemical industry as well as in food and cosmetic industries.

## Figures and Tables

**Table 1:** Percent concentration (%) of chemical constituents of *T. flousiana* stem bark essential oils.

No.	Compounds	Molecular Formula	Percentage (%)	RI ^a^	RI ^b^(Reference)	Methods of Identification
**Fatty Acids**						
1	Nonanoic acid	C_9_H_18_O_2_	0.03	1264	2192 [24]	a,c,d
2	Decanoic acid	C_10_H_20_O_2_	0.03	1361	2298 [25]	a,c,d
3	Dodecanoic acid	C_12_H_24_O_2_	0.18	1560	2503 [24]	a,c,d
4	Tridecanoic acid	C_13_H_26_O_2_	0.02	1657	2617 [25]	a,c,d
5	Tetradecanoic acid	C_14_H_28_O_2_	1.10	1764	2670 [25]	a,c,d
6	14-Pentadecenoic acid	C_15_H_28_O_2_	0.52	1848	3181 [26]	a,c,d
7	Pentadecanoic acid	C_15_H_30_O_2_	1.37	1868	2822 [25]	a,c,d
8	Palmitoleic acid	C_16_H_30_O_2_	0.81	1946	2948 [27]	a,c,d
9	9-Hexadecenoic acid	C_16_H_30_O_2_	0.09	1952	-	a,c,d
10	Hexadecanoic acid	C_16_H_32_O_2_	27.13	1992	2931 [25]	a,c,d
11	*cis*-10-Heptadecenoic acid	C_17_H_32_O_2_	0.08	2055	-	a,c,d
12	Heptadecanoic acid	C_17_H_34_O_2_	0.20	2068	2305 [28]	a,c,d
13	Linoleic acid	C_18_H_32_O_2_	13.48	2155	3157 [29]	a,c,d
14	α-Linolenic acid	C_18_H_30_O_2_	5.41	2160	3193 [29]	a,c,d
**Monoterpenes**						
15	α-Terpineol	C_10_H_18_O	0.03	1194	1706 [24]	a,c,d
16	Carvacrol	C_10_H_14_O	0.02	1301	2241 [30]	a,c,d
**Sesquiterpenes**						
17	(-)-Spathulenol	C_15_H_24_O	0.09	1599	2144 [25]	a,c,d
18	Widdrol	C_15_H_26_O	0.12	1611	2179 [31]	a,c,d
19	Cedrol	C_15_H_26_O	0.13	1614	2093 [32]	a,c,d
20	6-Methyl-2-(4-methylcyclohex-3-en-1-yl)hepta-1,5-dien-4-ol	C_15_H_24_O	0.05	1631	-	a,c,d
21	8-Cedren-13-ol	C_15_H_24_O	0.11	1637	2199 [33]	a,c,d
22	γ-Eudesmol	C_15_H_26_O	0.12	1640	2193 [24]	a,c,d
23	*epi*-α-Muurolol	C_15_H_26_O	0.06	1650	1621 [34]	a,c,d
24	β-Eudesmol	C_15_H_26_O	0.06	1660	2257 [24]	a,c,d
25	T-cadinol	C_15_H_26_O	0.13	1662	2187 [31]	a,c,d
26	Germacra-4(15),5E,10(14)-trien-1β-ol	C_15_H_24_O	0.05	1667	-	a,c,d
27	Humulenol-II	C_15_H_24_O	0.09	1681	-	a,c,d
28	α-Bisabolol	C_15_H_26_O	0.05	1688	2232 [35]	a,c,d
29	Longifolaldehyde	C_15_H_24_O	0.02	1692	-	a,c,d
30	β-Acoradienol	C_15_H_24_O	0.05	1787	-	a,c,d
31	Drimenol	C_15_H_26_O	0.04	1817	1772 [36]	a,c,d
**Diterpenes**						
**32**	Ambrial	C_16_H_26_O	0.07	1809	-	a,c,d
33	Biformene	C_20_H_32_	0.32	1937	1907 [37]	a,c,d
34	Cembrene	C_20_H_32_	0.16	1958	-	a,c,d
35	Manoyl oxide	C_20_H_34_O	0.64	2000	2376 [31]	a,c,d
36	13-*epi*-Manoyl oxide	C_20_H_34_O	0.11	2021	2335 [38]	a,c,d
37	Manool	C_20_H_3__4_O	0.03	2027	2180 [39]	a,c,d
38	Geranyl linalool	C_20_H_34_O	0.06	2033	1912 [37]	a,c,d
39	Abietatriene	C_20_H_30_	1.04	2063	2065 [37]	a,c,d
40	Thunbergol	C_20_H_34_O	0.02	2076	-	a,c,d
41	Abieta- 7, 13- diene	C_20_H_32_	0.04	2088	-	a,c,d
42	2-Penten-1-ol, 3-methyl-5-[octahydro-4,5-dimethyl-7a-(1-methylethenyl)-1H-inden-4-yl-	C_20_H_34_O	16.16	2121	-	a,c,d
43	Sandaracopimarinal	C_20_H_30_O	0.17	2192	-	a,c,d
44	Larixol	C_20_H_34_O_2_	0.28	2211	-	a,c,d
45	4-*epi*-Dehydroabietol	C_20_H_30_O	0.77	2227	-	a,c,d
46	Nimbiol	C_18_H_24_O_2_	0.26	2258	-	a,c,d
47	Isopimara-7,15-dien-3-one	C_20_H_30_O	0.26	2261	-	a,c,d
48	*trans*-Totarol	C_20_H_30_O	0.19	2286	2280 [32]	a,c,d
49	Dehydroabietal	C_20_H_28_O	0.78	2305	-	a,c,d
50	Podocarpa-6,8,11,13-tetraen-12-ol, 13-isopropyl-, acetate	C_22_H_30_O_2_	7.56	2332	-	a,c,d
51	Ferruginol	C_20_H_30_O	6.52	2339	2327 [37]	a,c,d
52	Hinokione	C_20_H_28_O_2_	0.51	2463	-	a,c,d
53	Dronabinol	C_21_H_30_O_2_	0.11	2515	-	a,c,d
**Esters**						
54	Methyl pentadecanoate	C_15_H_30_O_2_	0.22	1825	2099 [29]	a,c,d
55	Diisobutyl phthalate	C_16_H_22_O_4_	0.14	1870	-	a,c,d
56	Cyperolactone	C_15_H_22_O_2_	0.02	1873	2480 [40]	a,c,d
57	Methyl hexadecanoate	C_17_H_34_O_2_	0.46	1926	2226 [41]	a,c,d
58	Methyl linoleate	C_19_H_34_O_2_	0.35	2099	2490 [42]	a,c,d
59	Methyl linolenate	C_19_H_32_O_2_	0.17	2105	2478 [42]	a,c,d
**Phenols**						
60	2-Allyl-4-methylphenol	C_10_H_12_O	0.05	1373	-	a,c,d
61	2,2-Methylene-bis(4-methyl-6-tert-butylphenol)	C_23_H_32_O_2_	0.10	2421	-	a,c,d
**Alkanes**						
62	Docosane	C_22_H_46_	0.07	2200	2196 [34]	a,c,d
63	Pentacosane	C_25_H_52_	0.05	2499	2500 [25]	a,c,d
64	Heptacosane	C_27_H_56_	0.06	2698	2700 [25]	a,c,d
65	Nonacosane	C_29_H_60_	0.03	2895	2900 [25]	a,c,d
**Others**						
66	2,5,5,8a-Tetramethyl-4-methylene-6,7,8,8a-tetrahydro-4H,5H-chromen-4a-yl hydroperoxide	C_14_H_22_O_3_	0.02	1740	-	a,c,d
67	*cis*-9-Hexadecenal	C_16_H_30_O	0.03	1750	-	a,c,d
68	1-Hexadecanol	C_16_H_34_O	0.17	1881	2384 [27]	a,c,d
69	13-Heptadecyn-1-ol	C_17_H_32_O	0.03	2041	-	a,c,d
	Total		89.70			

a Kováts retention indices (RI) calculated from the retention time in relation to those of a series of C7–C30 n-alkanes on a HP-5 column. b Kováts retention indices (RI) on HP-Innowax column from literature. c Values compared with [43]. d The mass spectra of authentic reference compounds where possible and by reference to NIST 17 database. “-” Kováts retention indices (RI) on HP-Innowax column was not found from the literature.

**Table 2:** IC_50_ of *T. flousiana* essential oil and butachlor on the content of chlorophyll a, chlorophyll b, and total chlorophyll of *S. communis.*

Pigment	Treatment	Time (h)	Regression Equation	r	IC_50_ (μg/mL)	95%CL (μg/mL)
Chlorophyll a	Essential oil	24	y = 1.2251 + 1.9312x	0.9327	90.10	74.01–109.68
		48	y = −0.0821 + 3.0372x	0.9896	47.13	42.09–52.76
		72	y = 1.6596 + 2.0762x	0.9437	40.64	34.98–47.21
		96	y = 2.6742 + 1.3534x	0.9536	52.29	42.40–64.50
	Butachlor	24	y = 0.7963 + 2.4091x	0.9459	55.58	48.57–63.60
		48	y = 0.9345 + 2.5763x	0.9794	37.85	33.08–43.30
		72	y = 1.5056 + 2.2331x	0.9946	36.71	31.66–42.58
		96	y = 1.4714 + 2.2568x	0.9174	36.60	31.49–42.54
Chlorophyll b	Essential oil	24	y = 0.7342 + 2.1024x	0.9438	106.91	86.86–131.58
		48	y = −1.2225 + 3.7115x	0.9759	47.49	42.73–52.77
		72	y = 1.9149 + 1.7859x	0.9560	53.39	45.22–63.05
		96	y = 2.7763 + 1.2659x	0.9584	57.09	45.60–71.47
	Butachlor	24	y = 0.6332 + 2.3004x	0.9186	79.12	67.10–93.00
		48	y = −0.0687 + 2.8175x	0.9467	62.95	55.74–71.10
		72	y = 1.5328 + 2.0981x	0.9424	44.93	38.67–52.20
		96	y = 1.8578 + 1.8762x	0.9530	47.29	40.24–55.57
Total chlorophyll	Essential oil	24	y = 1.2976 + 1.9193x	0.9309	84.92	70.19–102.75
		48	y = −0.0821 + 3.0372x	0.9623	46.62	41.67–52.15
		72	y = 1.6945 + 2.2008x	0.9200	31.77	27.16–37.17
		96	y = 2.7412 + 1.3035x	0.9482	54.05	43.48–67.18
	Butachlor	24	y = 0.6609 + 2.4596x	0.9436	58.09	50.76–66.49
		48	y = 0.7453 + 2.6025x	0.9719	43.14	37.87–49.14
		72	y = 1.7028 + 2.0456x	0.9566	40.91	35.07–47.74
		96	y = 1.7113 + 2.0495x	0.9256	40.24	34.43–47.04

**Table 3:** IC_50_ *T. flousiana* essential oil and Butachlor on the content of chlorophyll a, chlorophyll b, and total chlorophyll of *S. communis* with or without light (96 h).

Treatment		Regression Equation	r	IC_50_ (μg/mL)	95%CL (μg/mL)
**With Light**	chlorophyll a	y = −1.9294 + 3.7360x	0.9062	71.58	57.66–88.84
	chlorophyll b	y = −1.5076 + 3.3476x	0.9310	87.89	67.42–114.59
	total chlorophyll	y = −2.0191 + 3.6402x	0.9473	84.77	65.94–108.97
**Without Light**	chlorophyll a	y = 2.1333 + 0.9279x	0.9814	1229.24	121.24–12463.23
	chlorophyll b	y = 1.6774 + 1.0713x	0.9071	1156.28	156.28–10205.66
	total chlorophyll	y = 2.1286 + 0.9302x	0.9328	1221.58	120.76–12357.62

**Table 4:** The antifungal activities of the essential oil of *T. flousiana* (EC_50_).

Fungus	Time (h)	Regression Equation	r	EC_50_ (μg/mL)	95%CL(μg/mL)
***Rhizoctonia solani***	24	y = 1.8819 + 1.2679x	0.9876	287.94	195.23–424.68
***Colletotrichum gloeosporioiles***	48	y = 2.8088 + 0.8498x	0.9261	378.90	205.68–698.17
***Fusarium moniliforme***	72	y = 2.4418 + 0.8627x	0.9865	923.03	529.24–1609.80
***Thanatephorus cucumeris***	72	y = 1.4164 + 1.2822x	0.9716	623.36	453.38–857.06
***Fusariun oxysporun***	72	y = 1.5112 + 1.1997x	0.9754	809.07	545.56–1199.86
***Didymella bryoniae***	72	y = 1.5316 + 0.9910x	0.9122	3162.34	1187.37–8422.33

**Table 5:** IC_50_ values of *T*. *flousiana* essential oil and ascorbic acid measured by 2,2-diphenyl-1-picrylhydrazyl (DPPH).

Treatment	Regression Eq	IC_50_ (μg/mL)	r	95% CL (μg/mL)
Essential oil	y = 1.6712 + 2.1903*x*	33.51	0.9766	26.15–42.95
Ascorbic acid	y = 3.5608 + 1.6001*x*	7.98	0.9808	5.76–11.04

All treatments were performed in triplicates and repeated at least three times. IC_50_: concentration (μg/mL) for a 50% inhibition.

## Sample Availability

Samples of the compounds are available from the authors.

## References

[1] Wei J.G., Zhou G., Liu F., Yang Z., Mo S.Z., He B. (2018). Accumulation and distribution pattern of carbon in ecosystems of *Taiwania flousiana* and successive rotations of *Cunninghamia lanceolata* plantations. Chin. Subtropical Agric. Res..

[2] Yin R.P., Lu D.W., Zhang C.H., Jiang Y., Yang D.J., Pu Y.Q. (2018). The biological characteristics of planted *Taiwania flousiana*. J. West. China For. Sci..

[3] Nie S. (1998). Studies on the pulping paper-making of *Taiwania flousiana*. Chin. J. Fujian Technol..

[4] Chen K. (2018). Cultivation Method of Taiwania flousiana with High Survival Rate and High Germination Rate. CN Patent.

[5] Lian Y.J., Lin X.S., Chen Y.P., Zhou Z.Z., Zhang X.D., Zheng Z.W., Mao S.P., Lin W.F., You F.L. (2017). Method for Raising Seedlings of Taiwania flousiana. CN Patent.

[6] Jiang F., Deng L., Liu L.L., Li F.S., Liu S.L., Lin X.H., Wang J.S. (2015). Non-Woven Lightweight Matrix Seedling Breeding Method of Taiwania flousiana. CN Patent.

[7] Huang C.B., Cao J.Z., Wu Q.B., Wei J.G., Meng Y.H., Li B.P. (2010). Comparative analysis on soil physi-chemical properties and the tree growth in *Taiwania flousiana* plantations and successive rotation plantations of *Cunninghamia lanceolata*. Scientia Silvae Sinicae.

[8] He B., Huang H.C., Cao M., Huang C.B., Meng Y.H., Rong Y., Chen Y.P. (2009). Concentration, accumulation and distribution of microelements in *Taiwania flousiana* plantation. Chin. J. Nanjing For. Univ..

[9] Xiang Y., Yang S.P., Zhan Z.J., Yue J.M. (2004). Terpenoids and phenols from *Taiwania flousiana*. Acta. Bot. Sin..

[10] Kagamizono T., Kyo T., Tanaka T., Maejima A., Arai Y., Sho S., Chin Y., Tei K. (1997). Lignans as Bone Resorption Inhibitors. JP Patent.

[11] Zhou L.J., Huang J.G., Li J.L. (2019). Herbicidal Plant Extract and Application the Extract of Taiwania flousiana. CN Patent.

[12] Lu C.M., Wu G.R., Tao M.X., Zhou C.F., Wei J.C. (1999). Effects of osmatic stress and paraquat on SOD in *Spirogyra communis*(Hassall) Kutzing. J. Plant Resour. Environ..

[13] Takano T., Higuchi S., Ikegaya H., Matsuzaki R., Kawachi M., Takahashi F., Nozaki H. (2019). Identification of 13 *Spirogyra* species (Zygnemataceae) by traits of sexual reproduction induced under laboratory culture conditions. Sci. Rep..

[14] Ajayi-Oyetunde O.O., Everhart S.E., Brown P.J., Tenuta A.U., Dorrance A.E., Bradley C.A. (2019). Genetic Structure of *Rhizoctonia solani* AG-2-2IIIB from Soybean in Illinois, Ohio, and Ontario. Phytopathology.

[15] Krishnan N., Velramar B., Velu R.K. (2019). Investigation of antifungal activity of surfactin against mycotoxigenic phytopathogenic fungus *Fusarium moniliforme* and its impact in seed germination and mycotoxicosis. Pestic. Biochem. Phys..

[16] Michielse C.B., Rep M. (2009). Pathogen profile update: *Fusarium oxysporum*. Mol. Plant Pathol..

[17] Skandamis P., Koutsoumanis K., Fasseas K., Gje N. (2001). Inhibition of oregano essential oil and EDTA on *Escherichia coli* o157:h7. Ital. J. Food Sci..

[18] Stević T., Berić T., Šavikin K., Soković M., Gođevac D., Dimkić I., Stanković S. (2014). Antifungal activity of selected essential oils against fungi isolated from medicinal plant. Ind. Crops Prod..

[19] Meepagala K.M., Schrader K.K., Wedge D.E., Duke S.O. (2005). Algicidal and antifungal compounds from the roots of *Ruta graveolens* and synthesis of their analogs. Phytochemistry.

[20] Ghosh S., Ozek T., Tabanca N., Ali A., Rehman J.U., Khan I.A., Rangan L. (2014). Chemical composition and bioactivity studies of *Alpinia nigra* essential oils. Ind. Crops Prod..

[21] Ambrosio C.M.S., Alencar S.M.D., Sousa R.L.M.D., Moreno A.M., Gloria E.M.D. (2017). Antimicrobial activity of several essential oils on pathogenic and beneficial bacteria. Ind. Crops Prod..

[22] Baczek K.B., Kosakowska O., Przybył J.L., Pióro-Jabrucka E., Costa R., Mondello L., Gniewosz M., Synowiec A., Weglarz Z. (2017). Antibacterial and antioxidant activity of essential oils and extracts from costmary (*Tanacetum balsamita* L.) and tansy (*Tanacetum vulgare* L.). Ind. Crops Prod..

[23] Harkat-Madouri L., Asma B., Madani K., Said Z.B.O.S., Rigou P., Grenier D., Allalou H., Remini H., Adjaoud A., Boulekbache-Makhlou L. (2015). Chemical composition, antibacterial and antioxidant activities of essential oil of *Eucalyptus globulus*, from Algeria. Ind. Crops Prod..

[24] Kivcak B., Mert T., Demirci B., Baser K.H.C. (2001). Composition of the essential oil of *Arbutus unedo*. Chem. Nat. Compd..

[25] Altintas A., Kose Y.B., Yucel E., Demirci B., Baser K.H.C. (2004). Composition of the essential oil of *Centaurea dichroa*. Chem. Nat. Compd..

[26] Kheder F.B.H., Mahjoub M.A., Zaghrouni F., Kwaja S., Helal A.N., Mighri Z. (2014). Chemical composition antioxidant and antimicrobial activities of the essential oils of *Matricaria aurea* Loefl. growing in Tunisia. J. Essent. Oil Bear. Pl..

[27] Kushnarenko S.V., Karasholakova L.N., Ozek G., Abidkulova K.T., Mukhitdinov N.M., Baser K.H.C., Ozek T. (2016). Investigation of essential oils from three natural populations of *Lonicera iliensis*. Chem. Nat. Compd..

[28] Pino J.A., Marquez E., Castro D. (2009). Volatile and non-volatile acids of noni (*Morinda citrifolia* L.) fruit. J. Sci. Food Agric..

[29] Senatore F., Formisano C., Rigano D., Piozzi F., Rosselli S. (2007). Chemical composition of the essential oil from aerial parts of *Stachys palustris* L. (Lamiaceae) growing wild in Southern Italy. Croat. Chem. Acta.

[30] Atti-Santos A.C., Pansera M.R., Paroul N., Atti-Serafini L., Moyna P. (2004). Seasonal variation of essential oil yield and composition of *Thymus vulgaris* L. (Lamiaceae) from South Brazil. J. Essent. Oil Res.

[31] Sezik E., Kocakulak E., Baser K.H.C., Ozek T. (2005). Composition of the essential oils of *Juniperus oxycedrus* SUBSP. *macrocarpa* from Turkey. Chem. Nat. Compd..

[32] Chéraif I., Jannet H.B., Hammami M., Khouja M.L., Mighri Z. (2007). Chemical composition and antimicrobial activity of essential oils of *Cupressus arizonica* Greene. Biochem. Syst. Ecol..

[33] Karik Ü., Çinar O., Tunçtürk M., Sekeroglu N., Gezici S. (2018). Essential oil composition of some sage (*Salvia spp*.) species cultivated in İzmir (Turkey) ecological conditions. Indian J. Pharm. Educ. Res..

[34] Lis A., Banaszczak P. (2011). Composition of the essential oil of *Phellodendron piriforme*. Chem. Nat. Compd..

[35] Baser K.H.C., Demirci B. (2004). The essential oil of *Senecio farfarifolius* Boiss. et Kotschy growing in Turkey. J. Essent. Oil Res..

[36] Linde J., Combrinck S., Vuuren S.V., Rooy J.V., Ludwiczuk A., Mokgalaka N. (2016). Volatile constituents and antimicrobial activities of nine South African liverwort species. Phytochem. Lett..

[37] Fkiri S., Ghazghazi H., Rigane G., Ben Salem R., Mezni F., Khaldi A., Khouja M.L., Nasr Z. (2019). Chemical compositions and biological activities essential oil from the needles of North African *Pinus Pinaster* Var. Rev. Roum. Chim..

[38] Öztürk B., Özek G., Özek T., Baser K.H.C. (2014). Chemical diversity in volatiles of *Helichrysum plicatum* DC. subspecies in Turkey. Rec. Nat. Prod..

[39] Boussaada O., Ammar S., Saidana D., Chriaa J., Chraif I., Daami M., Helal A.N., Mighri Z. (2008). Chemical composition and antimicrobial activity of volatile components from capitula and aerial parts of *Rhaponticum acaule* DC growing wild in Tunisia. Microbiol. Res..

[40] Clery R.A., Cason J.R.L., Zelenay V. (2016). Constituents of cypriol oil (*Cyperus scariosus* R.Br.): N-Containing molecules and key aroma components. J. Agr. Food Chem..

[41] Altintas A., Kosar M., Kirimer N., Baser K.H.C., Demirci B. (2006). Composition of the essential oils of *Lycium barbarum* and *L. ruthenicum* fruits. Chem. Nat. Compd..

[42] Boussaada O., Saidana D., Chriaa J., Chraif I., Ammar S., Mahjoub M.A., Mighri Z., Daami M., Helal A.N. (2008). Chemical composition and antimicrobial activity of volatile components of *Scorzonera undulata*. J. Essent. Oil Res..

[43] Adams R.P. (2007). Identification of Essential Oil Components by Gas Chromatography/Mass Spectrometry.

[44] Horvathova E., Navarova J., Galova E., Sevcovicova A., Chodakova L., Snahnicanova Z., Melusova M., Kozics K., Slamenova D. (2014). Assessment of antioxidative, chelating, and DNA-protective effects of selected essential oil components (eugenol, carvacrol, thymol, borneol, eucalyptol) of plants and intact *Rosmarinus officinalis* oil. J. Agric. Food Chem..

[45] Sarikurkcu C., Arisoy K., Tepe B., Cakir A., Abali G., Mete E. (2009). Studies on the antioxidant activity of essential oil and different solvent extracts of *Vitex agnus*-*castus* L. fruits from Turkey. Food Chem. Toxicol..

[46] Chang S.T., Cheng S.S., Wang S.Y. (2001). Antitermitic activity of essential oils and components from *Taiwania* (*Taiwania cryptomerioides*). J. Chem. Ecol..

[47] Chang S.T., Chen P.F., Wang S.Y., Wu H.H. (2001). Antimite activity of essential oils and their constituents from *Taiwania Cryptomerioides*. J. Med. Entomol..

[48] Miguel M.G. (2010). Antioxidant activity of medicinal and aromatic plants. A review. Flavour Frag. J..

[49] Gross E.M., Hilt S., Lombardo P., Mulderij G. (2007). Searching for allelopathic effects of submerged macrophytes on phytoplankton-state of the art and open questions. Hydrobiologia.

[50] Katarína K., Katarína K., Svajlenová O., Ján V. (2004). Biological activity of copper (II) N-salicylideneaminoacidato complexes. Reduction of chlorophyll content in freshwater alga *Chlorella vulgaris* and inhibition of photosynthetic electron transport in spinach chloroplasts. Chem. Pap..

[51] Han H., Chen Y., Jørgensen S.E., Nielsen S.N., Hu W. (2009). A system-dynamic model on the competitive growth between *Potamogeton malaianus* Miq. and *Spirogyra* sp. Ecol. Model..

[52] Zuo S., Zhou S., Ye L., Ma S. (2016). Synergistic and antagonistic interactions among five allelochemicals with antialgal effects on bloom-forming *Microcystis aeruginosa*. Ecol. Eng..

[53] Wang H., Liang F., Zhang L. (2015). Composition and anti-cyanobacterial activity of essential oils from six different submerged macrophytes. Pol. J. Environ. Stud..

[54] Wang H., Xi B., Cheng S., Wang Y., Zhang L. (2015). Phenolic and fatty acids from pomegranate peel and seeds: Extraction, identification and determination of their anti-algal activity. Fresen. Environ. Bull..

[55] Chen Y.H., Lin C.Y., Yen P.L., Yeh T.F., Cheng S.S., Chang S.T. (2017). Antifungal agents from heartwood extract of *Taiwania cryptomerioides*, against brown root rot fungus *Phellinus noxius*. Wood Sci. Technol..

[56] Wang S.Y., Wu J.H., Shyur L.F., Kuo Y.H., Chang S.T. (2002). Antioxidant activity of abietane-type diterpenes from heartwood of *Taiwania cryptomerioides* Hayata. Holzforschung.

[57] Ho C.L., Yang S.S., Chang T.M., Su Y.C. (2012). Composition, antioxidant, antimicrobial and anti-wood-decay fungal activities of the twig essential oil of *Taiwania cryptomerioides* from Taiwan. Nat. Prod. Commun..

[58] Ferreira M.J., Costantin M.B., Sartorelli P., Rodrigues G.V., Limberger R., Henriques A.T., Kato M.J., Emerenciano V.P. (2001). Computer-aided method for identification of components in essential oils by ^13^C NMR spectroscopy. Anal. Chim. Acta..

[59] Babushok V.I., Linstrom P.J., Zenkevich I.G. (2011). Retention indices for frequently reported compounds of plant essential oils. J. Phys. Chem. Ref. Data.

[60] Pacheco R., Ferreira A.F., Pinto T., Nobre B.P., Loureiro D., Moura P., Gouveia L., Silva C.M. (2015). The production of pigments & hydrogen through a *Spirogyra* sp. biorefinery. Energ. Convers. Manage..

[61] Dere S., Gunes T., Sivaci R. (1998). Spectrophotometric determination of chlorophyll-a, b and total carotenoid contents of some algae species using different solvents. Turk. J. Bot..

[62] Houot V., Etienne P., Petitot A., Barbier S., Blein J., Suty L. (2001). Hydrogen peroxide induces programmed cell death features in cultured tobacco BY-2 cells, in a dose-dependent manner. J. Exp. Bot..

[63] Boubaker H., Karim H., Hamdaoui A.E., Msanda F., Leach D., Bombarda I., Vanloot P., Abbad A., Boudyach E.H., Ait Ben Aoumar A. (2016). Chemical characterization and antifungal activities of four thymus, species essential oils against postharvest fungal pathogens of citrus. Ind. Crops Prod..

[64] CLSI (2006). Performance Standards for Antimicrobial Disk Susceptibility Tests.

[65] Verma S.K., Goswami P., Verma R.S., Padalia R.C., Chauhan A., Singh V.R., Darokar M.P. (2016). Chemical composition and antimicrobial activity of bergamot-mint (*Mentha citrata* Ehrh.) essential oils isolated from the herbage and aqueous distillate using different methods. Ind. Crops Prod..

[66] El-Gawad A.M.A. (2016). Chemical constituents, antioxidant and potential allelopathic effect of the essential oil from the aerial parts of *Cullen plicata*. Ind. Crops Prod..

